# Mathematical modelling of the pathogenesis of multiple myeloma-induced bone disease

**DOI:** 10.1002/cnm.2645

**Published:** 2014-05-09

**Authors:** Bing Ji, Paul G Genever, Ronald J Patton, Michael J Fagan

**Affiliations:** 1 School of Control Science and Engineering, Shandong University17923 Jingshi Road, Jinan, 250061, People's Republic of China; 2 Department of Biology, University of YorkHeslington, York, YO10 5DD, UK; 3 School of Engineering, University of HullCottingham Road, Hull, HU6 7RX, UK

**Keywords:** multiple myeloma, MM-induced bone disease, bone microenvironment, mathematical model, osteoblast and osteoclast activities

## Abstract

Multiple myeloma (MM) is the second most common haematological malignancy and results in destructive bone lesions. The interaction between MM cells and the bone microenvironment plays an important role in the development of the tumour cells and MM-induced bone disease and forms a ‘vicious cycle’ of tumour development and bone destruction, intensified by suppression of osteoblast activity and promotion of osteoclast activity. In this paper, a mathematical model is proposed to simulate how the interaction between MM cells and the bone microenvironment facilitates the development of the tumour cells and the resultant bone destruction. It includes both the roles of inhibited osteoblast activity and stimulated osteoclast activity. The model is able to mimic the temporal variation of bone cell concentrations and resultant bone volume after the invasion and then removal of the tumour cells and explains why MM-induced bone lesions rarely heal even after the complete removal of MM cells. The behaviour of the model compares well with published experimental data. The model serves as a first step to understand the development of MM-induced bone disease and could be applied further to evaluate the current therapies against MM-induced bone disease and even suggests new potential therapeutic targets. © 2014 The Authors. *International Journal for Numerical Methods in Biomedical Engineering* published by John Wiley & Sons Ltd

## 1 INTRODUCTION

Multiple myeloma (MM) is the second most frequent haematological malignancy, and MM-induced bone disease is a major cause of morbidity for MM patients 1. MM induces increased bone resorption and suppressed bone formation leading to a negative bone balance and osteolytic lesions that rarely heal 2,3. Histomorphometric studies reveal that the increased bone loss arises from enlarged bone resorption surfaces and deeper resorption depths at individual remodelling sites 4,5. In parallel, uncoupling between bone resorption and bone formation is also observed in MM patients 6.

The interaction between MM cells and the bone microenvironment (MM–bone interaction) plays an important role in the development of MM-induced bone disease. It promotes tumour growth and survival, as well as the consequent bone destruction 1. Recently, many biochemical factors have been implicated in the development of MM-induced bone disease, for example, cytokines with osteoclast activating function, such as the receptor activator of nuclear factor kappa-B ligand (RANKL), macrophage colony-stimulating factor, interleukin-6 (IL-6), IL-11 and IL-1*β* 7, which are produced or stimulated by MM–bone interaction and further stimulate osteoclast activation and proliferation, leading to increased bone resorption. In turn, growth factors released from bone resorption stimulate the growth of myeloma cells 5, including transforming growth factor-beta (TGF-*β*), bone morphogenetic proteins, heparin-binding fibroblast growth factors and insulin-like growth factor I 8,9. Such reciprocal interaction produces a vicious cycle between MM cells and the bone microenvironment, stimulating both tumour development and bone destruction 1,5.

Mathematical modelling has demonstrated great potential in aiding our understanding and analysis of complex biological systems, and several mathematical models of bone remodelling have been proposed in recent years to integrate our fragmented knowledge of the bone remodelling process 10–20. However, very few mathematical models have been constructed to simulate and investigate the development of MM-induced bone disease. As far as we are aware, currently, only two models have been developed to analyse the role of MM–bone interaction in the development of MM disease. Ayati *et al*. 21 proposed a model to simulate the dynamics of normal bone remodelling and MM disease. However, this model does not include the specific molecular mechanisms involved in the development of the MM-induced bone disease, and the model parameters lack corresponding biological meaning. Wang *et al*. 22 constructed another model to mimic MM–bone interaction and identify the signalling mechanisms that are believed to drive the progression of MM disease. This model includes IL-6 and signalling pathways involved in MM and bone marrow stromal cell (BMSC) adhesion. However, Wang *et al*. 22 do not consider the role of osteoblast inhibition and the antimyeloma effect of small leucine-rich proteoglycans (SLRPs, expressed by mature osteoblasts) in the development of the MM disease – but it is known that both are important in bone destruction and development of tumour cells in MM patients 2,7. Stimulation of osteoblast differentiation is thought to be able to reduce tumour burden and bone destruction in MM patients. Thus, drugs such as bortezomib, a boron-containing molecule with the potential to enhance osteoblast proliferation and bone formation in MM patients, have been proposed as a potential target for MM-induced bone disease 3,23. Similarly, interventions targeting SLRPs are also suggested as potential therapies for MM disease 2. Hence, inclusion of these mechanisms to allow the investigation of such potential management pathways is clearly essential and a key driver of the current work.

Osteoblast inhibition is caused primarily by the blockade of the differentiation of osteoblast precursors into mature osteoblasts, with secreted factors produced by MM cells and MM–bone interaction both resulting in the suppression of osteoblastic activity 3. The suppressed osteoblast activity not only increases the ratio of RANKL to osteoprotegerin (OPG), enhancing osteoclastogenesis and bone resorption, but also stimulates antiapoptotic factors and growth factors for MM cells, which form a positive feedback between osteoblast suppression and the growth of MM cells 1,3. Importantly, several potential therapies against MM disease target the disease's suppression of osteoblastic activity, such as bortezomib-related therapy 3 and inhibition of TGF-*β* 2. In this paper, a mathematical model of the interaction between the MM cells and the bone microenvironment is described. It was developed in parallel with the recently published model of Wang *et al*. 22, being similarly based on the earlier work of Pivonka *et al*. 14, but unlike the model of Wang *et al*. 22, it also includes the underlying mechanisms of osteoblast inhibition and its role in the development of MM-induced bone disease. The model can simulate the development of MM and the induced bone destruction and explains why MM-induced bone lesions rarely heal even after the complete removal of MM cells. It is based on our current knowledge of the pathogenesis of MM, which inevitably will increase, but the model can easily be refined and improved as more data become available.

## 2 MODEL DEVELOPMENT

### 2.1 Basic structure of the model

The bone microenvironment consists of many different components including multiple cell types and matrix proteins. The contribution of each component of the bone microenvironment to the progress and survival of tumour cells is still not completely understood 1,3. However, it is certain that the suppression of osteoblast activity and the enhancement of osteoclast activity are both key factors in the development of tumour cells and the bone destruction 2,7.

**Figure 1:**
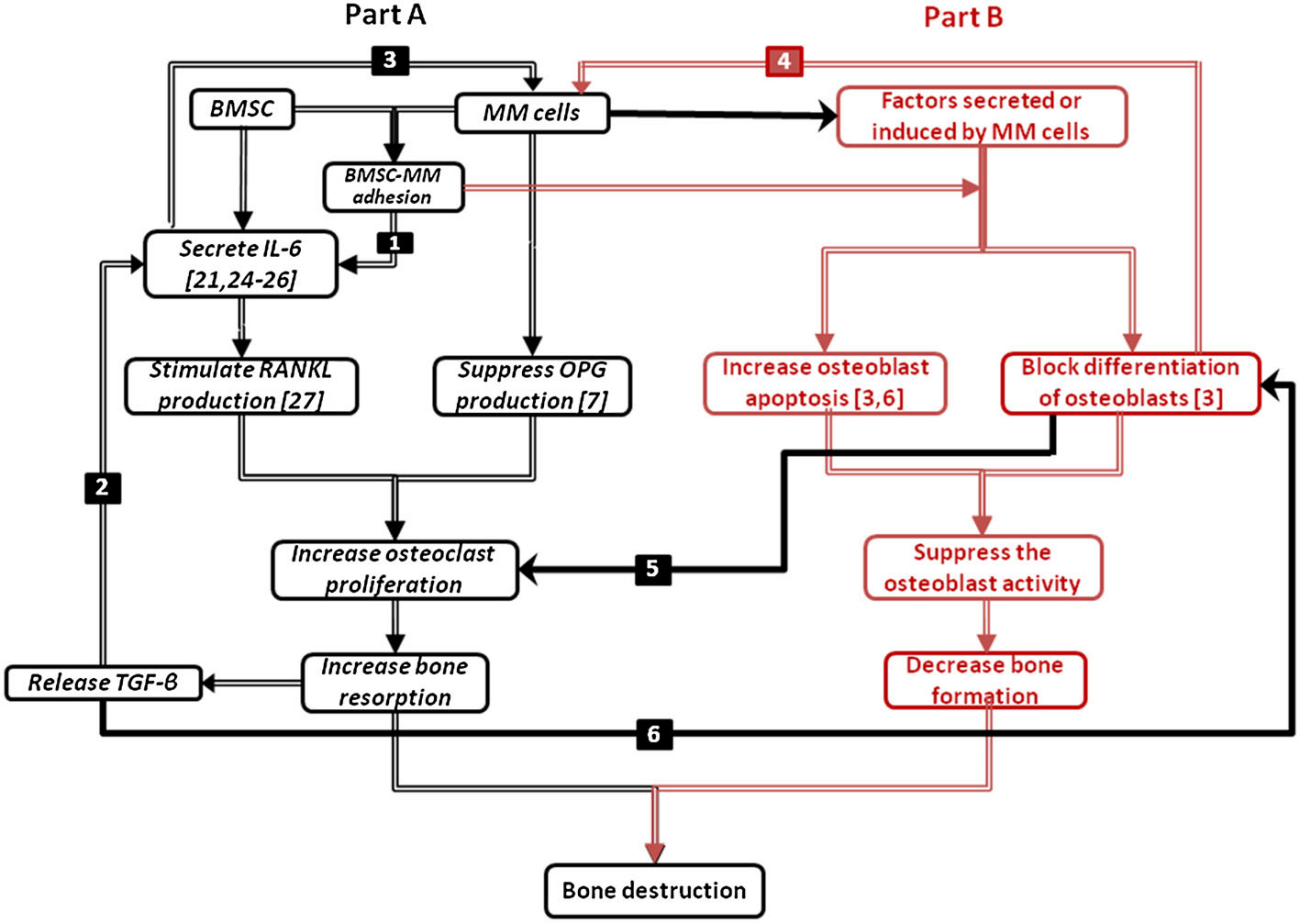
Proposed cellular interactions in multiple myeloma (MM) development. (1) Bone marrow stromal cell (BMSC)-MM cells adhesion enhances the production of interleukin-6 (IL-6) by BMSCs 24; (2) transforming growth factor-beta (TGF-*β*) stimulates the production of IL-6 25,26; (3) IL-6 stimulates the proliferation of MM cells 25–27; (4) immature osteoblasts support the growth and survival of MM cells, whereas mature osteoblasts enhance the apoptosis of MM cells; (5) the blockade of differentiation into mature osteoblasts contributes to the increase of the ratio of receptor activator of nuclear factor kappa-B ligand/osteoprotegerin (RANKL/OPG) and thus promotes osteoclasts proliferation; (6) and TGF-*β* potentially inhibits later phases of osteoblast differentiation and maturation. For further information on the significance of the different colours and solid/hollow connecting lines, see the main text.

The basic structure of the model proposed here is shown in Figure1 and demonstrates the vicious cycle in MM disease, with the appearance of MM cells changing the bone microenvironment, resulting in osteolysis, which in turn promotes the proliferation of further MM cells 5. The model structure consists of two parts: part A (in black and connected in black hollow connecting lines) is associated with osteoclasts and the bone resorption aspects of the disease, whereas part B (in red and connected in red solid connecting lines) deals with osteoblasts and bone formation activities. (Note that, for simplicity, Figure1 does not include the direct interactions between osteoblastic and osteoclastic lineages. These mechanisms are well described in literature [e.g. 14], but for convenience, they are summarised in FigureA1 in Appendix A).

Part A describes how MM cells increase bone resorption, which in turn stimulates the proliferation of MM cells. Here, two positive feedback cycles exist. Firstly, IL-6 secreted by BMSC stimulates the production of RANKL by osteoblast precursors 28, while MM cells suppress the production of OPG by mature osteoblasts 7. Consequently, the increased RANKL-OPG ratio promotes bone resorption 7. In turn, TGF-*β* released from bone matrix by the bone resorption stimulates the secretion of IL-6 by BMSC 25,26, where the IL-6 production can also be enhanced by BMSC-MM cell adhesion 20. Secondly, IL-6 and BMSC-MM cell adhesion promotes the proliferation of MM cells, which in turn further stimulates IL-6 production and BMSC-MM cell adhesion 24,25,27.

Part B describes the reciprocal relationship between the suppression of osteoblastic activity and the stimulation of MM cell production. Both BMSC-MM cell adhesion and secreted factors (produced or induced by MM cells) can block the differentiation of BMSCs into mature osteoblasts and at the same time stimulate osteoblast apoptosis, which inhibits osteoblast activity and resultant bone formation 3,6,29–31. On the other hand, the blockade of differentiation into mature osteoblasts can stimulate MM cell production, because immature osteoblasts support growth and survival of myeloma cells, whereas mature osteoblasts enhance apoptosis of myeloma cells 2. Thus, in the underlying mechanism, IL-6 secreted by immature osteoblasts (BMSCs) promotes MM cell growth and resistance to apoptosis 32, whereas matrix components such as SLRPs, including decorin, are expressed mature osteoblasts and have an antimyeloma effect 33.

Parts A and B also have direct connections with each other (the interaction between parts A and B are marked in solid black connecting lines in Figure1), that is, the blockade of differentiation into mature osteoblasts contributes to an increase in the RANKL/OPG ratio, because RANKL is produced primarily by immature osteoblasts, whereas OPG is produced primarily by mature osteoblasts (marked as black solid arrow no. 5 in Figure1) 14,34. In addition, TGF-*β* released by bone resorption inhibits osteoblast activity, because TGF-*β* potentially inhibits later phases of osteoblast differentiation and maturation (marked as black solid arrow no. 6 in Figure1) 2.

### 2.2 Model equations

The model equations are mathematical representations of the basic mechanisms and relationships shown in Figure1. Differentiation into active osteoclasts and osteoblasts from their progenitors involves several intermediate stages. For example, as many as seven stages have been identified for osteoblastic differentiation from BMSCs to osteocytes and bone lining cells 35, whereas the osteoclast lineage develops from haematopoietic precursor cells through monocyte differentiation and fusion to osteoclast formation 36,37. Here and following Pivonka *et al*. 14, four stages of osteoblastic differentiation (uncommitted progenitors (BMSCs); osteoblasts precursors; active osteoblasts; and osteocytes, bone lining cells or apoptotic osteoblasts) and three stages of osteoclastic differentiation (osteoclast precursors, active osteoclasts and apoptotic osteoclasts) are considered in our model, with three stages of MM cells (MM cell precursors, active MM cells and apoptotic MM cells).

Thus, overall, the proposed model contains four state variables: osteoblast precursors (OB_p_), active osteoblasts (OB_a_), active osteoclasts (OC_a_) and active MM cells (MM). ‘Hill functions’ are used to represent the cellular interaction via the single ligand to receptor binding and are denoted by *π* functions 14. Thus, Equations (m1) and (m2) denote the stimulating and inhibiting functions of the ligand-receptor binding respectively, where ‘L’ represents the concentration of the ligand, ‘β’ represents maximal expression level of the promoter, ‘n’ is the coefficient that regulates the steepness of the function ‘*π*’ and ‘k_1_’ and ‘k_2_’ represent dissociation constants. To ensure consistency with Pivonka *et al*. 14, both ‘β’ and ‘n’ are both assumed to equal 1.

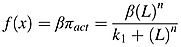
(1)

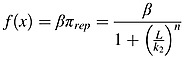
(2)

Using the same nomenclature as Pivonka *et al*. 14 for convenience, the equations describing the dynamics of cell concentrations are then proposed as follows:

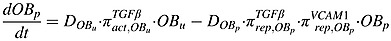
(3)

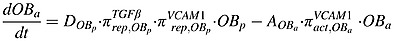
(4)

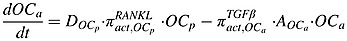
(5)

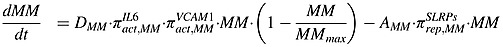
(6)where *OB*_*p*_,  *OB*_*a*_, *OC*_*a*_ and *MM* represent concentrations of osteoblast precursors, active osteoblasts, active osteoclasts and active MM cells, respectively, and 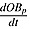 is the variation of *OB*_*p*_ with time, for example. Similarly, *OB*_*u*_ and *OC*_*p*_ are concentrations of uncommitted osteoblastic progenitors and osteoclastic precursors and are set as constants in the model, because their populations are relatively large. 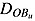, 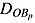, 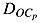 and *D*_*MM*_ represent the differentiation rates of uncommitted osteoblast progenitors, osteoblast precursors, osteoclast precursors and MM cell precursors, respectively, and 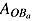, 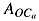 and  *A*_*MM*_ are apoptosis rates of active osteoblasts, active osteoclasts and active MM cells, respectively. *MM*_*max*_ is the maximum concentration of MM cells. The production of MM cells is regulated by several secreted factors, such as IL-6, insulin-like growth factor 1, vascular endothelial growth factor and macrophage inflammatory protein-1 1,5,7,22. *D*_*MM*_ represents the proliferation of MM cells regulated by IL-6 and BMSC-MM cell adhesion. Note that under normal/healthy conditions without MM cells, the terms 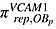 and 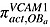 still exist in Equations (m3) and (m4), because vascular cell adhesion molecule 1 (VCAM-1, expressed on BMSCs) is always present 7,25. Pivonka *et al*. 14 ignored the effect of VCAM-1 in their formulation, and hence, the behaviour of the two models differs under normal conditions.

The RANK-RANKL-OPG pathway plays an important role in the regulation of osteoclast activity, and RANKL can stimulate osteoclastogenesis by binding to RANK on the osteoclast progenitors, while RANKL-mediated osteoclastogenesis is inhibited by OPG, a soluble decoy receptor for RANKL 38. Growth factors, such as TGF-*β*, released during bone resorption can stimulate osteoblast recruitment and the migration and proliferation of osteoblast precursors 39–41, while inhibiting production of mature osteoblasts. As in the model of Pivonka *et al*. 14, 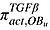, 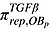, 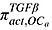 and 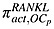 represent the effect of TGF-*β* and RANKL on osteoclastic and osteoblastic lineages. Thus, 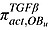 represents the stimulation of uncommitted osteoblastic progenitors into osteoblastic precursors, 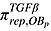 represents the inhibition of the differentiation of osteoblastic precursors into active osteoblasts, 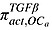 represents the promotion of the apoptosis of active osteoclasts by TGF-*β* and 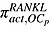 reflects the fact that RANKL produced by osteoblastic precursors stimulates the differentiation of osteoclastic precursors into active osteoclasts. 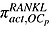 also includes OPG secreted by active osteoblasts inhibiting the differentiation osteoclastic precursors, by binding to RANK expressed on osteoclastic precursors. According to the proposed forms of the Hill functions in Equations (m1) and (m2), the *π* functions involving TGF-*β* and RANKL are defined as follows:

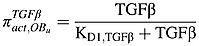
(7)

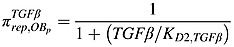
(8)

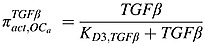
(9)

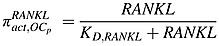
(10)where

- TGF-*β* and *RANKL* represent the concentrations of TGF-*β* and RANKL, respectively, and their definitions are included in Tables1 and 2, and
- the definitions and values of *K*_*D*1,*TGFβ*_, *K*_*D*2,*TGFβ*_,  *K*_*D*3,*TGFβ*_ and *K*_*D*,*RANKL*_  are included in Table3.

**Table I:** Definitions of the concentrations of RANKL, OPG, TGF-*β*, PTH, IL-6, SLRPs, VLA-4 and VCAM-1.

*RANKL*	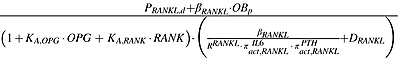
*OPG*	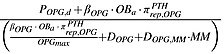
*TGFβ*	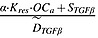
*PTH*	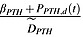
*IL*6	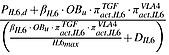
*SLRPs*	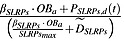
*VLA*4	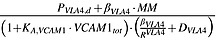
*VCAM*1	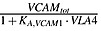

These definitions are derived on the basis of similar principles to those described in Pivonka *et al*. 14.

RANKL, receptor activator of nuclear factor kappa-B ligand; OPG, osteoprotegerin; TGF-*β*, transforming growth factor-beta; PTH, parathyroid hormone; IL-6, interleukin-6; SLRP, small leucine-rich proteoglycan; VLA-4, very late antigen-4; VCAM-1, vascular cell adhesion molecule 1.

**Table II:** Definitions of the *π* functions used in the concentration equations in Table1.

PTH stimulates the production of RANKL	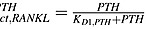
PTH inhibits the production of OPG	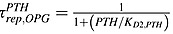
IL-6 stimulates the production of RANKL	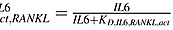
VAL-4 stimulates the production of IL-6	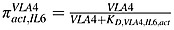
*TGF* − *β* stimulates the production of IL-6	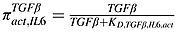
SLRPs produced by mature osteoblasts suppresses the proliferation of MM cells	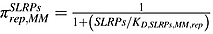

RANKL, receptor activator of nuclear factor kappa-B ligand; OPG, osteoprotegerin; TGF-*β*, transforming growth factor-beta; PTH, parathyroid hormone; IL-6, interleukin-6; SLRP, small leucine-rich proteoglycan; VLA-4, very late antigen-4; MM, multiple myeloma.

**Table III:** Definitions and values of model parameters used in the model of multiple myeloma-induced bone disease.

Parameters	Description	Value
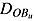	Differentiation rate of osteoblast progenitors	3.24e + 2/day (estimated)
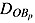	Differentiation rate of osteoblast precursors	3.67e-1/day (estimated)
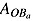	Rate of elimination of active osteoblasts	3.00e-1/day 14
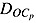	Differentiation rate of osteoclast precursors	1.73e-1/day (estimated)
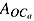	Rate of elimination of active osteoclasts	1.20/day 14
*K*_*D*1,*TGFβ*_	Activation coefficient related to growth factors binding on *OB*_*u*_	4.28e-4 pM (calculation by GA)
*K*_*D*2,*TGFβ*_	Repression coefficient related to growth factors binding on *OB*_*p*_	2.19e-4 pM (estimated)
*K*_*D*3,*TGFβ*_	Activation coefficient related to growth factors binding on *OC*_*a*_	4.28e-4 pM 14
*K*_*D*1,*PTH*_	Activation coefficient for RANKL production related to PTH binding	2.09e + 1 pM (calculation by GA)
*K*_*D*2,*PTH*_	Repression coefficient for OPG production related to PTH binding	2.21e-1 pM 14
*K*_*D*,*TGFβ*,*IL*6,*act*_	Half-maximal concentration of TGF-*β* on promoting the production of IL-6	1.2e-4 pM (calculation by GA)
*K*_*D*,*IL*6,*RANKL*,*act*_	Half-maximal concentration of IL-6 on promoting the production of RANKL	0.2 pM (calculation by GA)
*K*_*D*,*RANKL*_	Activation coefficient related to RANKL binding to RANK	4.12e + 1 pM (estimated)
*α*	TGF-*β* content stored in bone matrix	1.00 pM/% 14
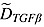	Rate of degradation of TGF - *β*	2.00e + 2/day 42
β_PTH_	Rate of synthesis of systemic PTH	9.74e + 2 pM/day 43
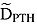	Rate of degradation of PTH	3.84e + 2/day 43
β_IL6_	Rate of synthesis of IL - 6 per cell	1.20e + 7/day^27^,^44^
D_IL6_	The degradation rate of IL-6	4.99e + 1/day 45
IL6_max_	The maximum concentration of IL-6	8.04e-1 pM 46
β_OPG_	Minimum rate of production of OPG per active osteoblast	5.02e + 6/day (estimated)
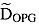	Rate of degradation of OPG	4.16/day 25
OPG_max_	Maximum possible OPG concentration	7.98e + 2 pM 65
β_RANKL_	Production rate of RANKL per cell	8.25e + 5/day (estimated)
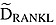	Rate of degradation of RANKL	4.16/day 47
*R*^*RANKL*^	Maximum number of RANKL on the surface of each osteoblastic precursor	3.00e + 6 14
RANK	Fixed concentration of RANK	1.28e + 1 pM 14
*K*_*A*,*OPG*_	Association rate constant for RANKL binding to OPG	5.68e-2/pM 48
*K*_*A*,*RANK*_	Association rate constant for RANKL binding to RANK	7.19e-2/pM 48
*K*_*res*_	Relative rate of bone resorption (normalised with respect to normal bone resorption)	2.00e + 2%/(pM day) 49
*K*_*form*_	Relative rate of bone formation (normalised with respect to normal bone resorption)	3.32e + 1%/(pM day) (calculation by GA)
*D*_*MM*_	MM proliferation controlled by IL-6 and BMSC-MM adhesion	5.50e-2/day (estimated)
*A*_*MM*_	Rate of elimination of active MM cells	2.00e-3/day 50
*MM*_*max*_	Maximum possible MM concentration	1.98 pM 51
*K*_*D*,*VCAM*1,*MM*,*act*_	Half-maximal concentration of VLA - 4 on promoting the MM cells production	1.5667e-4/pM (calculation by GA)
*K*_*D*,*VLA*4,*IL*6,*act*_	Half-maximal concentration of VLA - 4 on promoting the IL-6 production	1.88e + 4/pM (calculation by GA)
*K*_*D*,*IL*6,*MM*,*act*_	Half-maximal concentration of IL - 6 on promoting the MM cells production	1.2151e-5 pM (calculation by GA)
*K*_*D*,*SLRPs*,*MM*,*rep*_	Half-maximal concentration of SLRPs on promoting the MM cells production	1.306e + 9 pM (calculation by GA)
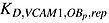	Half-maximal concentration of VCAM - 1 on repressing the differentiation of *OB*_*p*_	1.4e-1 pM (calculation by GA)
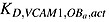	Half-maximal concentration of VCAM - 1 on promoting the apoptosis of *OB*_*a*_	2.2e-1 pM (calculation by GA)
β_VLA4_	Rate of synthesis of VLA - 4 per cell	2.04e + 6/day (estimated)
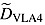	Rate of degradation of VLA-4	1.5/day (estimated)
*R*^*VLA*4^	Maximum number of VLA-4 expressed on the surface of MM cells	5.6e + 4 52
VCAM1_tot_	Total concentration of VCAM-1	1.92 pM 52
K_A,VCAM1_	The association rate for VLA-4 binding to VCAM-1	8.3e-2/pM 53
D_OPG,MM_	The degradation rate of OPG by MM cells	4.16/(pM day) (estimated)

RANKL, receptor activator of nuclear factor kappa-B ligand; OPG, osteoprotegerin; TGF-*β*, transforming growth factor-beta; PTH, parathyroid hormone; IL-6, interleukin-6; SLRP, small leucine-rich proteoglycan; VLA-4, very late antigen-4; VCAM-1, vascular cell adhesion molecule 1; MM, multiple myeloma; GA, genetic algorithm.

In Equation (m6), 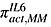 represents IL-6 regulation of the proliferation of MM cells. MM–bone interaction is carried out through the binding of CAMs, such as very late antigen-4 (*α*4*β*1 integrin present on the surface of MM cells) to VCAM-1, which is expressed on BMSC 7, and 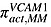 is used to represent the effect of MM-BMSC on the proliferation of MM cells. The underlying mechanism of MM-BMSC adhesion regulating the osteoblast lineage is complicated. It inhibits osteoblast activity by reducing the activity and expression of runt-related transcription factor 2, a critical transcription factor for osteoblast differentiation 3. For simplicity, 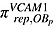 represents BMSC-MM cell adhesion that blocks the differentiation of mature osteoblasts from their progenitors, whereas 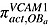 represents BMSC-MM cell adhesion stimulating the apoptosis of osteoblasts, and 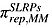 represents SLRPs produced by mature osteoblasts suppressing the proliferation of MM cells 3. The definitions of these *π* functions are as follows:

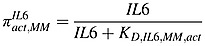
(11)

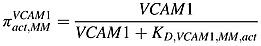
(12)

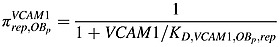
(13)

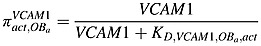
(14)

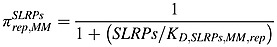
(15)where *IL*6, *VCAM*1 and *SLRPs* represent the concentrations of *IL*6, *VCAM*1 and *SLRPs*, respectively, and their definitions are included in Tables1 and 2. The definitions and values of the other parameters in Equations (11)–(15) are included in Table3.

Note that in Equations (m3), (m4) and (m6), the osteoblast lineage and MM cells are regulated by two ligands simultaneously, which are incorporated here through the multiplication of their respective Hill functions. Other, for example, additive, approaches are equally possible, and the sensitivity of the results to these different formulations should ideally be compared in the future. It should also be noted that secreted factors produced by MM cells may also suppress osteoblast differentiation by inhibiting Wnt signalling 2,3 but are not currently considered in the model because the underlying mechanisms are not completely clear 54,55. Thus, here, we assume that the effects of these and other secreted factors are minor compared with that of BMSC-MM cell adhesion. This is a limitation of the model.

The model for the bone resorption and formation activities is proposed as follows:

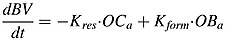
(16)where *BV* represents the normalised bone volume; *K*_*res*_ and *K*_*form*_ are the relative bone formation and resorption rates, respectively (their values are also included in Table3), and 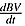 represents the variation of bone volume with time.

## 3 SIMULATION RESULTS AND DISCUSSION

In the absence of MM cells, the model (defined by the aforementioned equations) is able to replicate the behaviour of ‘healthy’ bone in that osteoblast and osteoclast interactions are governed by the relationships shown in FigureA1 in Appendix A, and the simulation predicts the correct steady-state cell populations and bone volume. This is discussed and demonstrated in detail in 56; the same information is therefore not repeated here. The bone microenvironment is always found to remain in a dynamic steady state, as do other biological systems under physiological conditions without external stimuli, and is able to return to a steady state after perturbations are removed 13,53. The model is used here to simulate how cell concentrations fluctuate from the steady state because of the invasion of MM cells but then return to the steady state after their removal. The variation in bone volume with time is also calculated to demonstrate the MM-induced bone destruction, and then, the reason for the bone destruction is examined by considering the variation in the ratio of *OB*_*a*_ to *OC*_*a*_. Also, a sensitivity study is reported to investigate how variations in the key model parameters (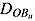, 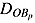, 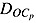, 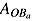, 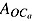, *A*_*MM*_, *β*_OPG_, *β*_RANKL_, *β*_PTH_, *β*_IL-6_ and 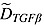) affect MM concentration and bone volume. Such studies allow the contributions of the different factors to be investigated and in the future might consider combinations of parameters and thereby allow potential targets for new therapies to be identified. The initial values of cell concentrations used in the model are listed in Table4.

**Table 4:** The initial values of cell concentrations in the model.

Variables	Values	Unit
*OB*_*u*_	3.27e-6 [57–58]	pM
*OB*_*p*_	7.67e-4 [19]	pM
*OB*_*a*_	6.39e-4 [59–60]	pM
*OC*_*p*_	1.28e-3 [61]	pM
*OC*_*a*_	1.07e-4 [59–60]	pM
MM	3.26e-1 [51, 62]	pM

MM cell concentration is at day 201; other cell concentrations are at day 1.

In the model, any unknown parameters (i.e. those parameters where experimental data are unavailable or those that have no direct biological meaning) may be calculated via a genetic algorithm (GA) as summarised in Table3. Thus, because a parameter may be directly or indirectly related with one or more of the initial values of cells concentrations listed in Table4 (e.g. 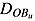 and 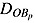 involve the experimental data of the initial concentration of *OB*_*p*_ in Table4), these initial values are set as targets for the parameter fitting. The calculation of the model parameters is then achieved by trying different values in a domain and then picking those that provide the best fit with corresponding experimental data. On the basis of these values, the remaining unknown model parameters are then calculated according to relevant experimental data through GA. Thus, the GA approach effectively considers all possible combinations of the unknown parameters and predicts the optimal values, taking many hours on a powerful PC and potentially considering billions of combinations in its search for the optimum set. Although the accuracy of its predictions of the unknown parameters obviously cannot be checked, it does avoid the inevitable trial and error and/or guesswork involved in otherwise ‘estimating’ the values. The simulation was carried using the Matlab computational software package (v7.7.0, Mathworks, Natick, USA).

### 3.1 Simulation of multiple myeloma-induced bone disease

**Figure 2:**
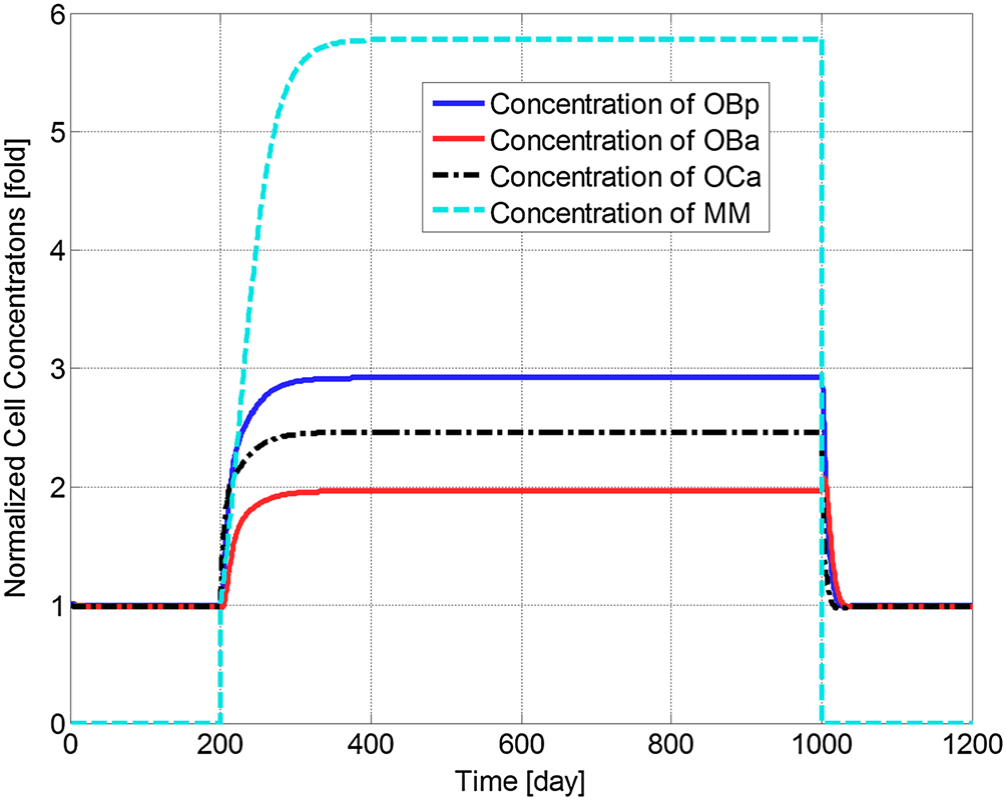
Model simulations of the normalised variation in the concentrations of osteoblast precursors, active osteoblasts, active osteoclasts and active tumour cells with respect to their respective initial values (multiple myeloma (MM) cells are injected at day 201 and removed at day 1001).

**Figure 3:**
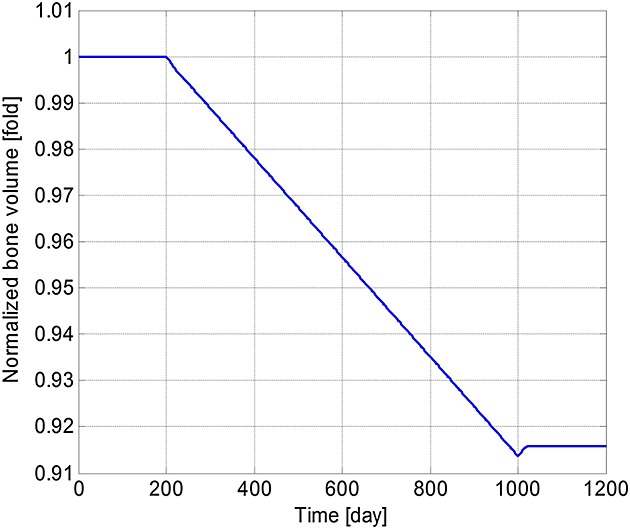
Model simulations of the variation in the normalised bone volume with respect to its initial value (multiple myeloma cells are injected at day 201 and removed at day 1001).

**Figure 4:**
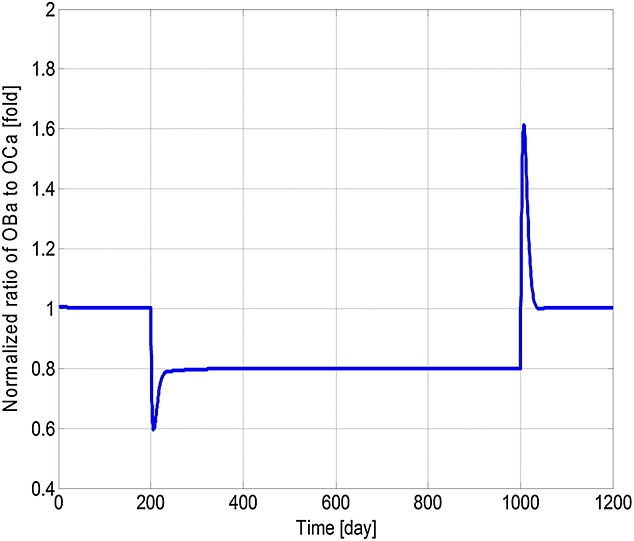
Model simulations of the variation in the normalised ratio of active osteoblasts to active osteoclasts with respect to the initial ratio (multiple myeloma cells are injected at day 201 and removed at day 1001).

**Figure 5:**
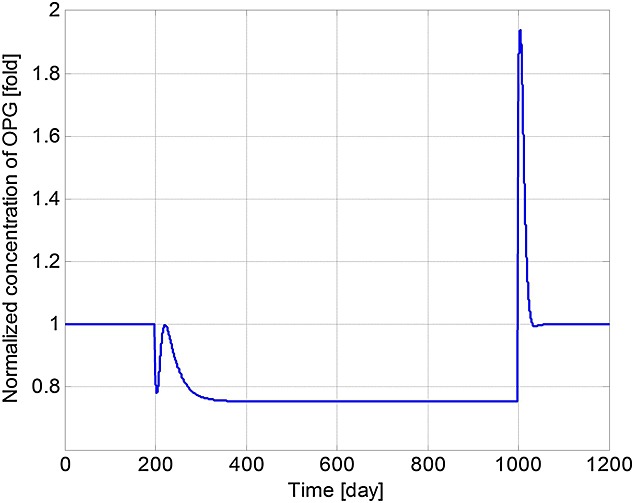
Model simulations of the variation in normalised osteoprotegerin (OPG) concentration with respect to its initial value (multiple myeloma cells are injected at day 201 and removed at day 1001).

**Figure 6:**
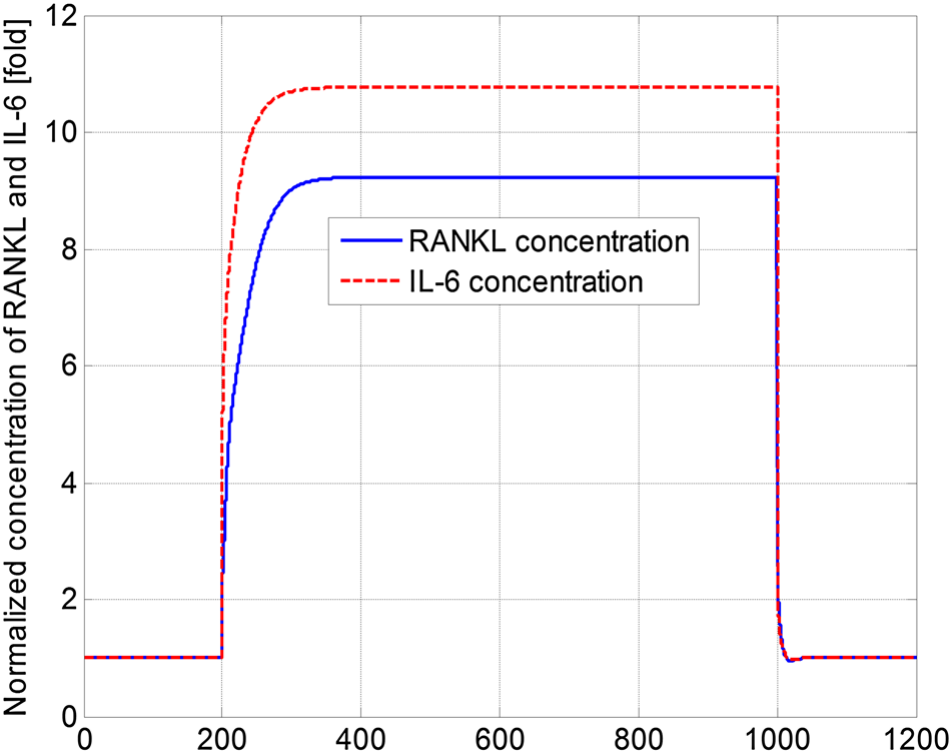
Model simulations of the variation in normalised receptor activator of nuclear factor kappa-B ligand (RANKL) and interleukin-6 (IL-6) concentrations with respect to their initial values (multiple myeloma cells are injected at day 201 and removed at day 1001).

Figure2 confirms that the bone microenvironment remains in a steady state until the invasion of the MM cells, with cell concentrations constant at their initial values as given in Table4. The steady state is disturbed due to the appearance of MM cells after the 200th day, causing a fluctuation of cell concentrations, as illustrated in Figure2. Thus, *OB*_*p*_ concentration is seen to increase nearly threefold due to the invasion of MM cells, which arises because the MM cells inhibit the differentiation of *OB*_*p*_ into *OB*_*a*_ 3,29–31. The increase in concentrations of *OB*_*a*_,  *OC*_*a*_ and MM cells after the introduction of MM cells agrees with the experimental observations of Alexandrakis *et al*. 63, Diamond *et al*. 64 and Terpos *et al*. 65. The MM cell concentration increases to 578% of its original value, which is similar to the 600% increase reported in the experimental work of Diamond *et al*. 64. Figure3 confirms that the invasion of MM cells leads to bone destruction, which also agrees with the observation of a decline in bone volume in MM patients by Diamond *et al*. 64 and can be explained by the variation in the ratio of *OB*_*a*_ to *OC*_*a*_ as shown in Figure4. In addition, after the invasion of MM cells, OPG concentration decreases to 75% of that in the healthy condition (Figure5), which again compares well with experimental data ranging from 59% to 82% 65–68. Similarly, the increase in the IL-6 concentration to 1077% (shown in Figure6) is consistent with the 979% increase reported by Alexandrakis *et al*. 46. Also, RANKL concentration increased to 924% (also shown in Figure6), which again is within the observed range of experimental data: 226% 69 to 1567% 70.

It can be seen that some cell concentrations and the ratio of *OB*_*a*_ to *OC*_*a*_ undergo a short period of oscillation and then return to their initial steady-state values after the removal of tumour cells (Figures2, 4 and 5), which agrees with the observation that the steady state of biological systems is dynamic, and after the removal of external perturbations, they are capable of restoring themselves to the steady state again 13,71. The MM-induced bone destruction also stops after removal of the tumour cells; however, the bone volume remains at a lower level compared with its initial volume as shown in Figure3. This is because the ratio of *OB*_*a*_ to *OC*_*a*_ returns to its initial steady-state value after removal of the MM cells (Figure4), so that a near zero bone balance is achieved at the end of each subsequent remodelling cycle. This is consistent with the observation that MM-induced bone lesions rarely heal even after the removal of MM cells 3,7.

### 3.2 Sensitivity to the model parameters

**Figure 7:**
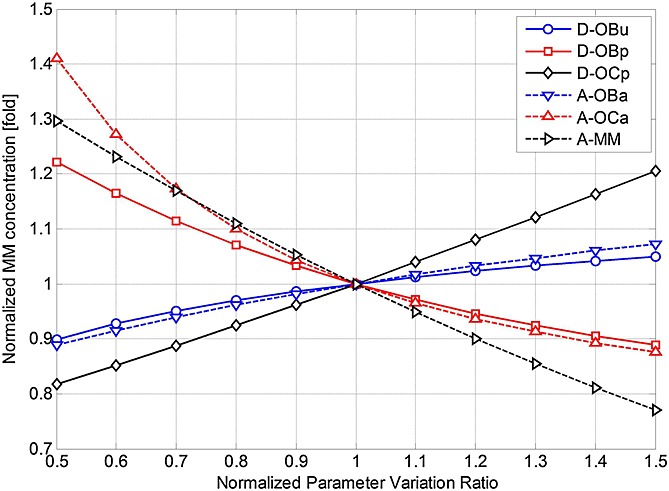
The effects of independently varying each model parameter (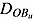, 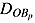, 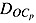, 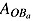, 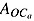 and *A*_*MM*_) on multiple myeloma (MM) concentration at day 1000. Parameter variance and MM concentration are normalised to the values of the base case.

**Figure 8:**
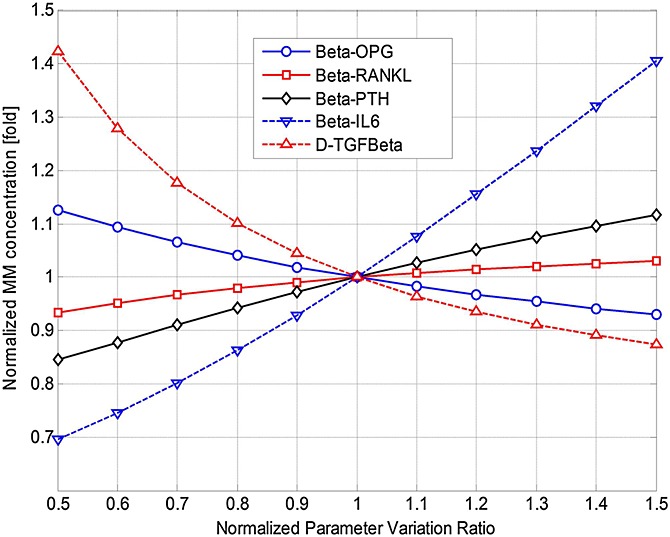
The effects of independently varying each model parameter (*β*_OPG_, *β*_RANKL_, *β*_PTH_, *β*_IL-6_ and 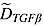) on multiple myeloma (MM) concentration at day 1000. Parameter variance and MM concentration are normalised to the values of the base case.

**Figure 9:**
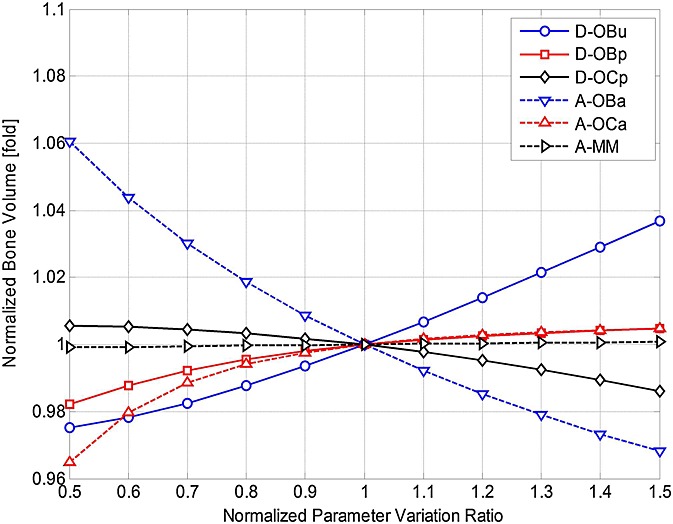
The effects of independently varying each model parameter (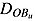, 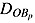, 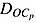, 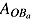, 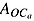 and *A*_*MM*_) on bone volume at day 1000. Parameter variance and bone volume are normalised to the values of the base case.

Further information on the underlying biochemical mechanisms are elucidated by the sensitivity study of 11 of the key parameters of the model (namely, 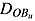, 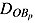, 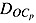, 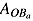, 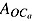, *A*_*MM*_, *β*_OPG_, *β*_RANKL_, *β*_PTH_, *β*_IL-6_ and 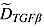), thereby suggesting possible strategies for management of MM. The parameters are varied individually between 50% and 150% of their initial base values (as defined in Table3), and the effects on MM concentrations and bone volume are examined, normalised with respect to their (maximal) values at day 1000 (in Figures2 and 3). Thus, Figures7 and 8 demonstrate how the variation in each parameter influences the maximum MM concentration at day 1000, and Figures9 and 10 show how bone volume is affected.

**Figure:**
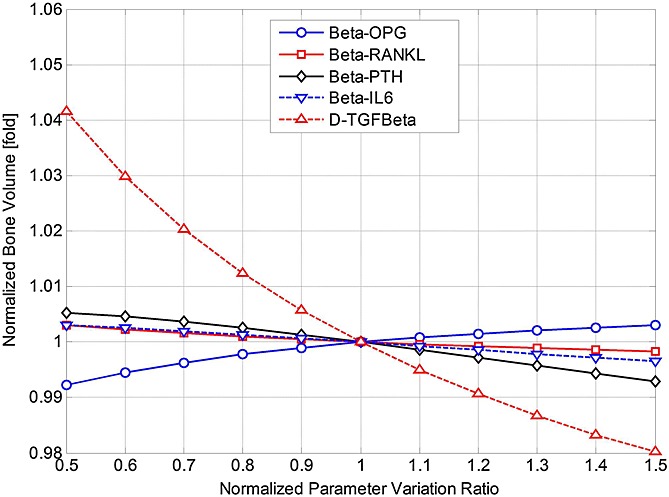
The effects of independently varying each model parameter (*β*_OPG_, *β*_RANKL_, *β*_PTH_, *β*_IL-6_ and 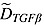) on bone volume at day 1000. Parameter variance and bone volume are normalised to the values of the base case.

Figures7 and 8 show that many of these 11 parameters have a significant influence on MM concentration. As some parameter values increase (between 50% and 150% of their base values), MM concentration increases, whereas the opposite effect is observed with the other parameters. For example, as *D_OCp_* increases from 50% to 150% of its base value, MM concentration varies by 81–121%. Conversely, for the same variation in *A_OCa_*, a significant decrease in MM concentration (from 141% to 87% of its base value) is observed. Figures9 and 10 show that this variation in parameter values affects bone volume. For example, a change in *A_OBa_* and 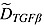 (from 50% to 150% of base value) produces a variation in bone volume (between 106% to 97% and 104% to 98%, respectively), whereas the same variation in *A*_*MM*_ has a negligible effect. The variations in *D_OCp_* and *A_OCa_* (from 50% to 150% of its base value) cause a decrease (between 101% and 99%) and an increase (between 96.5% and 100.5%) in bone volume, respectively.

## 4 CONCLUSION

In this paper, a model is proposed that simulates the interaction between MM cells and the bone microenvironment, and the contribution of that interaction to the progression of the MM cells and the resultant bone destruction. The development of MM-induced bone disease involves many biochemical factors and mechanisms, and most papers published to date have only considered part of those biochemical factors and mechanisms. The model in this paper integrates these partial findings and tries to analyse the progression of MM-induced bone disease comprehensively. It goes further than the recently published model of Wang *et al*. 22 by considering the key role of osteoblast inhibition and the antimyeloma effect of SLRPs in the development of the MM disease. Osteoblasts play an essential role in the development of MM bone disease, because their inhibition not only enhances osteoclastogenesis and bone resorption but also stimulates antiapoptotic factors and growth factors for MM cells. Thus, our model provides a more complete picture on how the equilibrium of the bone microenvironment is disturbed by the invasion of MM cells and then restored after their removal.

It should be noted that the effects of soluble factors responsible for inhibiting osteoblast activity are not considered in the current model. The model also only describes the temporal characteristics of the bone microenvironment, with no reference to spatial variations; it is also a population-based model rather than patient specific. As our knowledge of the pathogenesis of MM increases and we gain a better understanding of the key model parameters, it should be possible to further refine the model and integrate new findings and possibly move towards a patient-specific analysis.

In the meantime, the model demonstrates how bone cell concentrations fluctuate after the invasion of MM cells and how these variations result in bone destruction. The simulation results agree with published experimental data and explain why the lesions resulting from MM-induced bone destruction rarely heal even after the disappearance of MM cells. A sensitivity study is conducted to show how the variations in model parameters influence MM concentration and bone volume and thereby suggests potential treatment options for MM-induced bone disease. For example, the sensitivity study indicates that *D_OCp_* and *A_OCa_* are tightly related to MM concentration and bone volume. Thus, an intervention targeting these two factors could be a potential treatment for reducing the tumour burden. Indeed, bisphosphonate treatment for management of MM-induced bone disease does just that, by inhibiting the differentiation of osteoclast precursors into mature osteoclasts and promoting osteoclast apoptosis 72,73.

It is hoped that this paper will serve a first step to a more detailed analysis and understanding of the development of MM-induced bone disease. In the future, the model will be used to test and evaluate the efficacy of current therapeutic interventions for MM-induced bone disease, such as bisphosphonate and bortezomib, and inhibition of TGF-*β* and even propose new, more effective therapies for MM-induced bone diseases.

## APPENDIX

**Figure A1:**
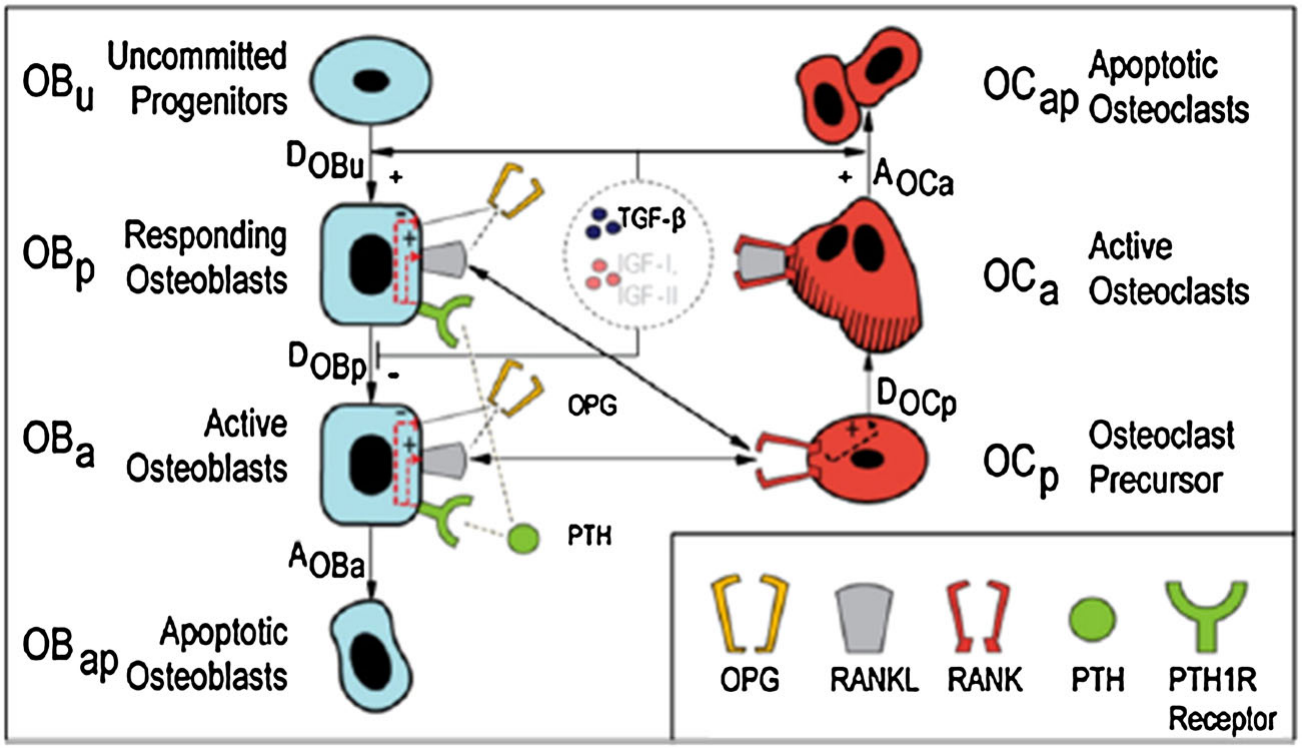
Schematic representation of the basic structure of interaction between osteoclastic and osteoblastic lineages. Reproduced from Pivonka *et al*. 14.
